# Complete Mesogastric Excisions Involving Anatomically Based Concepts and Embryological-Based Surgeries: Current Knowledge and Future Challenges

**DOI:** 10.3390/curroncol28060413

**Published:** 2021-11-22

**Authors:** Sergii Girnyi, Marcin Ekman, Luigi Marano, Franco Roviello, Karol Połom

**Affiliations:** 1 Department of Surgical Oncology, Medical University of Gdansk, 80-070 Gdansk, Poland; girnyisergii@gmail.com (S.G.); eq.marcin.ekman@gmail.com (M.E.); polom.karol@gmail.com (K.P.); 2 Unit of General Surgery and Surgical Oncology, Department of Medicine, Surgery and Neurosciences, University of Siena, 53100 Siena, Italy; franco.roviello@unisi.it

**Keywords:** mesogastrium, embryological planes, anatomical dissection, gastric cancer

## Abstract

Surgeries for gastrointestinal tract malignancies are based on the paradigm that we should remove the tumour together with its lymphatic drainage in one block. This concept was initially proposed in rectal surgery and called a total mesorectal excision. This procedure gained much interest and has improved oncological results in rectal cancer surgery. The same idea for mesogastric and complete mesogastric excisions was proposed but, because of the complexity of the gastric mesentery, it has not become a standard technique. In this review, we analysed anatomical and embryological factors, proposed technical aspects of this operation and incorporated the available initial results of this concept. We also discussed analogies to other gastrointestinal organs, as well as challenges to this concept.

## 1. Introduction

Gastric cancer is the fourth leading cause of death in worldwide cancer-associated deaths [1]. It is often diagnosed in the advanced stage. Treatment of patients with advanced gastric cancer still has poor prognoses [2]. Currently, the primary treatment for locally advanced gastric cancer is surgery. The standard surgical treatment for advanced gastric cancer worldwide is a gastrectomy with a D2 lymphadenectomy [3,4,5]. Rohatgi et al. have shown that despite radical surgical treatment, disease recurrence is observed in approximately 60% of patients [6].

The primary aim of gastrointestinal cancer surgery is an en block resection of the tumour together with its complex lymphovascular drainage that follows an organ’s specific mesenteric layers [7]. The concept of an en block resection of the untouched fascia covering the mesenteric tissue mimicking an envelope is the basis for complete meso-excisions. Heald et al. proposed a revolution in rectal surgery by implementing a total mesorectal excision [8]. This procedure involves a sharp dissection following embryological planes with intact mesorectal fascia that covers not only the tumour but also lymphatic vessels and regional lymph nodes. Following this idea, the local recurrence rate in conventional surgery, which was as high as 33%, dropped after the implementation of TME to 10% [9]. A similar idea was proposed by Hochenberger et al. for colon cancer [10]. A complete mesocolic excision with a dissection of the mesocolic planes together with vascular ligation led to an improved short survival rate in a German group and in a Danish population-based study [11]. The idea of applying mesentery-based surgeries for a number of other organs became popular and was subsequently proposed. A meso-oesophageal resection of the thoracic oesophagus was proposed by Matsubara et al., while Cuesta et al. presented a minimally invasive meso-oesophageal model [12,13].

The idea of the mesopancreas was presented by Adham and Singhirunnusorn, and a recent study presented robotic mesopancreatic resections on 289 patients [14]. In all, with an increasing number of centres implementing this idea together with clinical data into daily practices, this technique has already shown huge potential in this area [14,15]. 

The idea of meso-excision was also proposed in gastric cancer resections. 

Gastric cancer has four main routes of metastasis. These include a direct invasion of the tumour, lymphatic metastases, hematogenous metastases and peritoneal metastases. In 2012, Xie et al. hypothesized there may be a fifth pathway for the spread of gastric cancer cells called the metastasis V route [16]. This pathway differs from the other four classical metastatic pathways and cannot be removed by a standard D2 gastrectomy. Isolated tumour cells and small tumour nodules situated in the mesogastrium in adipose connective tissue have no direct link to the primary tumour or to lymphatic or vascular vessels. They proposed a third principle of radical gastrectomy, which is a complete mesogastrium excision (CME) [16]. As a new concept, this hypothesis needs further evidence, with preclinical as well as clinical studies, to become well established. In 2015, Xie et al. demonstrated the existence of the gastric mesentery and its structure. A model of the relationship between the stomach and the gastric mesentery surrounded by the proper fascia was then proposed. Previously unknown at that time, there are actually six anatomical structures formed between the embryological stage and the mature adult, and they consist of adipose tissue, lymph nodes and vessels. These help to fix the stomach to the posterior abdominal wall and were discovered and identified histologically. A CME using the Table Model technique and a tri-junction access was applied on a group of 105 patients to improve the conceptualization of gastric mesenteries [17]. The CME can also be called a systemic mesogastric excision or perigastic mesogastrium excision [18,19,20]. The CME was proposed by Xie et al.; however, a simple mesentery-based surgery that was the key to success in colorectal surgery is not that easy to implement during a gastrectomy because of various anatomical restrictions.

We need to point out here that, as with every new hypothesis, we need more evidence and detailed technical aspects of operation followed by CME principles to show that en block resection might be not enough in terms of oncological outcome. The primary and preliminary aspects of this new technique are presented and discussed. 

## 2. Mesogastrium

Due to the complexity of its structure, the mesogastrium is an anatomically unclassified structure. During embryological development, the tubular stomach and duodenum are connected to the posterior abdominal wall by a continuous mesentery. This mesentery consists of a double layer of peritoneum that surrounds the vessels, nerves and lymphatic pathways. As it grows and unfolds, the stomach begins to expand and twist to the left, clockwise around its longitudinal axis. At the same time, the pancreas, which is also a mesenteric component, arises from primitive buds in the duodenal wall. It then grows into the mesoduodenum and spleen, which is formed at the end of the first month of life, in the dorsal mesentery of the stomach, near its greater curvature, which is when it begins to enlarge [21,22]. 

During embryological development, both the stomach and duodenum are suspended by dorsal and ventral mesenteries from the parietal wall. During a 90-degree clockwise rotation along its longitudinal axis, the expansion of the dorsal mesogastrium into the upper abdomen occurs and is responsible for the formation of the omental bursa, which is fixed to the retroperitoneum. At the same time, there is a 270-degree counter-clockwise rotation of the primary midgut loop. The axis of rotation is the superior mesenteric artery. The transverse mesocolon moves closer to the dorsal mesogastrium. Both structures, the mesoduodenum and transverse mesocolon, are covered by the greater omentum, creating a derivative of the dorsal mesogastrium [23]. The creation of the mesopancreas is also an important part of the development of embryological planes and is important during mesogastric procedures in mesentery-based surgery for gastric neoplasms [23,24]. 

**Figure 1:**
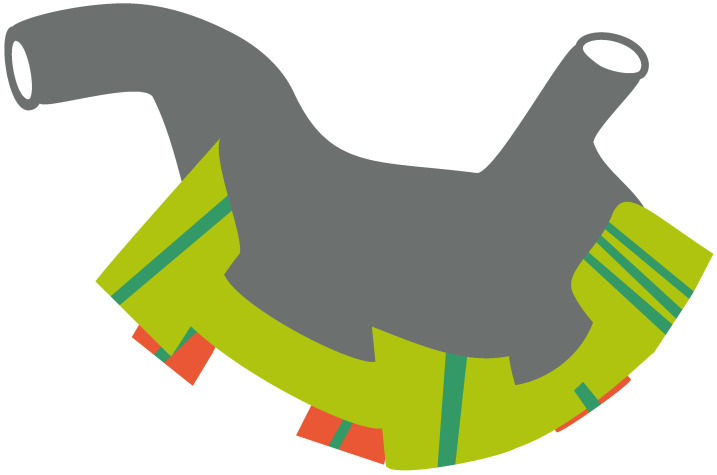
Table Model for complete mesogastric excision.

Xie et al. suggested that the mesentery of the stomach separates in three places into the greater mesentery and smaller mesentery of the stomach, the mesentery of the greater or lesser curvature of the stomach and the pancreatic mesentery. In the course of further embryological development, the mesentery of the stomach is divided into six independent areas. To date, no boundaries have been defined between the perigastric adipose tissue and the connective soft tissue. In 2015, Xie et al. reported the existence and described the anatomical structure of the gastric mesentery. They divided the mesogastrium into the following six segments: the left and right gastroepiploic mesenteries, the left and right gastric mesenteries, the posterior gastric mesenteries (PGM) and the short gastric mesenteries. We presented an idea of the Table Model in Figure 1. Each segment has its own artery. They also showed that the structure of each of the six mesogastrium segments varies according to their length and complexity [17]. Jie et al. hypothesized that the risk of cancer recurrence depends on the length and complexity of the mesentery [18]. Colorectal cancer recurrences are more frequent in sections with longer and more complex mesenteries, such as the hepatic and splenic flexures and the sigmoid colon, than in the remaining parts of the colon [25]. The possible mechanisms of the metastasis V route have been discussed in a publication by Xie et al.; briefly, we will describe the key points of this concept [16]. The surface of the stomach and connective and adipose tissues of the mesentery are covered by a layer of deep fascia. Within this space, tumour nodules may spread beyond the classic way of metastasis. In advanced gastric cancer, after a muscular layer invasion, cancer cells may drop into this envelope. The migration of cancer cells in this space is attracted by some cytokines, e.g., vistafin or DAB2IP [26,27,28]. This concept has been evaluated on cross-sectional analyses of the mesogastrium [29]. In a group of 40 patients with early gastric cancer, metastasis V was found in 2.5% of cases and in 24% in a group of 34 patients with advanced cancer. This type of metastasis was found at a mean distance of 2.6 cm from the gastric wall. Not only were the prognoses of the patients presenting metastasis V significantly worse, but this factor was also associated with other poor prognostic factors, such as tumour invasion depth and the involvement of several metastatic lymph nodes. 

### 2.1. Mesogastrium: Translation of the Idea from the Mesocolon

Shinohara et al. have shown similarities between the mesogastrium and mesocolon based on their series of patients [24]. In a group of 157 mesosigmoid specimens, they reported similarities in the allocation of lymph nodes in three sectors of the mesocolon. These mainly included the peri-organ, the intermediate and a root in comparison with mesogastric lymph node stations 1–6; the second sector, including stations 7, 8 and 10–12; and the last third in sector 9. The number of lymph nodes by sectors decreases as per the convergence and represents 36.5 for the stomach, 18 and 4 for the abovementioned sectors and 16, 7 and 4 for the analogous sectors in the mesocolon. A lower proportion in the main nodes originating in the mesogastrium might be associated with the shorter length of the celiac trunk when compared with the inferior mesenteric artery. 

### 2.2. Mesogastric Excision

In each segment of the mesogastric Table Model, various structures are situated, and different lengths and complexities are found between the segments [30]. There is a difference in length between the lesser and greater curvature mesenteries, with a much shorter length at the lesser mesentery and the upper part of the stomach; however, both shorter mesenteries are much more complex. A D1 + lymphadenectomy, according to Kumamoto et al., should be performed following the rules of a systemic mesogastric excision [19]. This group divided the D1 + lymphadenectomy for gastric cancer into four parts. The first part (the greater curvature segment) starts with a division of the greater omentum to open the bursa 3 cm from the gastroepiploic vessels up to the spleen. The left gastroepiploic vessels are then ligated at their roots. In case of a total gastrectomy, the additional dissection of the short gastric arteries up to the left cardia is performed. The second part (infrapyloric segment) of the dissection is between the mesoduodenum and greater omentum as well as the transverse colon. Infrapyloric lymph nodes are separated from the pancreas by following the intramesenteric dissectible layers. The third part (suprapancreatic and lesser curvature) starts with a dissection of the lesser curvature from the hepatoduodenal ligament up to the right cardia. The tissue with lymph nodes is separated from the intramesenteric dissectible layers. At this point, the tissues are separated from the arteries, including the proper hepatic, common hepatic and splenic arteries, and from the pancreas. Part four consists of a ligation of the right and left gastric vessels at their roots. In a total gastrectomy, we enlarge the dissection of lymph node stations 12a and 11d with what seems to be a simple extension of the narrowed part of the mesogastrium [19]. Similar steps in a laparoscopic D2 lymphadenectomy using the CME concept have been presented by Cao et al.; however, they started by lifting the stomach upward and in a cephalic direction by the assist [31]. The suprapancreatic mesogastrium, which consists of the left gastric mesentery (LGM), the right gastric mesentery (RGM) and the PGM, is then exposed. Opening the serosal layer and identifying the retrogastric space starts with the exposition of the LGM. Then, a tissue separation of the gastroduodenal artery (GDA) from the duodenal side is performed exposing the RGM. Afterwards, the LGM mobilization and the removal of tissue at the common hepatic artery (CHA) are performed. This manoeuvre helps in finding the root of the left gastric artery that is also ligated at its origin. Dissection and ligation of the RGM is performed along the CHA and portal vein. The superior border of the splenic vessels is then cleaned from the adjacent tissues. The anterior lobe of the PGM is then lifted to find and ligate the posterior gastric vessels. The mesogastric Table Model. 

## 3. Recurrence of GC after a D2 Gastrectomy

Surgical treatment with peri-operative chemotherapy is currently the only available method of treating advanced gastric cancer according to the current standards. The gold standard of radical surgical treatment of advanced gastric cancer is a total gastrectomy with a D2 lymphadenectomy. Unfortunately, a recurrence of the disease after radical surgery has been frequently reported [32,33,34,35,36,37]. The main causes of relapse are the potential spread of cancer cells during surgery or a minimal amount of cancer cells that have been left behind. Earlier studies have shown that cancer nodules in the mesogastrium can be as high as 8% [38]. Spolverato et al. stated that the recurrence rate of advanced gastric cancer is approximately 30–45% [39]. Rohatgi et al. have shown that, despite radical surgical treatment, disease recession was observed in approximately 60% of patients with advanced gastric cancer [6]. Likewise, Dickson et al. declared that, after radical surgery, 75–80% of patients had disease recurrence after two years [37]. Extra-nodal metastasis of cancer cells in adipose tissue was found in almost 40% of patients after a gastrectomy for T4a gastric cancer [40]. Broken dissected vessels during a gastric cancer lymphadenectomy may be responsible for cancer cell spillage into the peritoneal cavity [41].

## 4. Gastrectomy D2 + CME

A modification of the standard D2 gastrectomy to perform a CME was proposed by Xie et al. with a procedure appropriately named the Table Model [17]. We presented a modified scoring of the mesogastrium—amended proposition by Xie et al. [17] in Table 1. 

After standard gastrectomy with a D2 lymphadenectomy, the recurrence rate during the five years after the gastrectomy may be as high as 38%. Hypothetically, free cancer cells spill into the operation area and peritoneal cavity, which may be responsible for the cancer recurrence [42]. Xie et al. examined two groups of patients who had undergone either the classical D2 or D2 + CME laparoscopic distal gastrectomies. In particular, they looked at the intraoperative peritoneal washing before and after the operation and identified CEA levels to show the presence of gastric cancer cells [43]. From the group with a low CEA expression before the operation, they found that after the resection, 32% of CEA expression was in the peritoneal fluid and 15% was from the D2 + CME procedure. Additionally, in the group of patients with a low CEA expression, the DFS in the D2 + CME group was significantly better than in the D2 gastrectomy group. They postulated that cancer cells found in the peritoneum came from lymphatic vessels or from the serosal surface when it is involved in the cancer process. However, this hypothesis cannot support the situation when free cancer cells found in the peritoneal cavity also occur in tumours without a serosal invasion or without lymph node involvement [44,45]. The possible presence of cancer cells in the mesogastrium with no direct connection with the original tumour may be a possible explanation for intraperitoneal recurrence after a traditional gastrectomy with a D2 lymphadenectomy.

Short-term results after a laparoscopic D2 lymphadenectomy with CME on a group of 54 patients not only presented the feasibility of this procedure but also resulted in reduced blood loss, a good number of retrieved lymph nodes and improved short-term surgical outcomes [18]. Short-term outcomes in a randomized clinical trial on D2 + CME by Xie et al. suggested that this procedure, in comparison with the standard D2 dissection, exhibits a reduction in blood loss, more lymph nodes being able to be dissected and earlier postoperative flatus. Moreover, even as postoperative complications were comparable between the two groups, the severity was significantly lower in the D2 + CME group. A review of the available six studies on complete mesogastric excisions on 518 patients showed that the mean number of resected lymph nodes was 36.7 ± 10 [46]. The mean operative time was 240.7 ± 10.1 min, with a morbidity rate of 6% and a median blood loss of 50.2 ± 39.6 mL. The length of stay was 10.7 ± 0.7 days. These studies all came from China and Japan [18,19,20,31,47,48]. 

## 5. Challenges

### 5.1. Resection of the Pancreas

It has been shown, based on molecular and embryological points of view, that the pancreas is a separate mesenteric component arising from the duodenum. This process is controlled by the *pdx1* and *ptf1a* genes [21,49]. It has been shown that a pancreas-preserving total gastrectomy is not related with a higher recurrence rate and that lymphatic vessels do not pass through the pancreatic tissue. An adequate D2 lymphadenectomy close to the pancreatic tissue seems to be the optimal choice for radical procedures in the mesogastrium. Moreover, sparing the pancreatic tissue may be associated with a reduction in possible complications. 

### 5.2. Omentum and Peritoneal Lining of the Pancreas and Transverse Mesocolon

All the above-mentioned structures have been shown to be places where lymphatic channels link with the stomach. Additionally, these regions are well-recognized places of possible cancer cell implantation [50]. The idea of an omento-bursectomy has been a standard in Japanese gastrectomies since 1950. This radical approach was revisited after a phase III clinical trial (JCOG1001) showing that a bursectomy in resectable cT3/4a gastric cancer did not improve survival over a non-bursectomy [51]. Current Japanese guidelines still recommend an omentectomy in cT3/4 gastric cancer; however, its necessity is also under debate. Several studies have shown no difference in survival rates between an omentectomy and no omentectomy [52,53,54]. In a recent systematic review, Marano et al. reported no statistically significant prognostic difference in terms of overall survival between the bursectomy versus non-bursectomy groups [55]. Conversely, the resection of the bursa omentalis was associated with better overall survival than non-bursectomy surgery in serosa-positive gastric cancer patients.

It is important to say that, from embryonic anatomy, both the greater and lesser omentum are not authentic parts of the mesogastrium. They do not meet two main attributes of the mesogastrium, which include being located along the edge of the organ and encompassing some of the main blood vessels that connect them to the main organ. This is why, in the step-by-step resections previously mentioned, the omentum should be divided 3 cm from the edge of the gastroepiploic vessels. These are important parts of the mesogastric concept, and future analyses should also identify these aspects to show the hypothesis of the mesogastrium. 

### 5.3. Tailored Lymphadenectomy

An extended lymphadenectomy in colon cancer cases and a complete mesocolic excision has been adopted in many centres [10]. The extent of the lymphadenectomy following the idea of the mesocolon changes according to the position of the colon cancer. For a hepatic flexure or proximal transverse colon cancer, not only the lymph nodes of the mesentery but also the right gastroepiploic artery may need to be dissected for a complete peripancreatic lymph node dissection. Some have even advocated for the dissection of the nodes at least 10 cm from the right gastroepiploic vessels together with the subpyloric station and lymph nodes above the pancreatic head [56]. For cancer situated in the distal transverse colon as well as in the splenic flexure, additional lymph nodes from the inferior pancreas and along the left gastroepiploic artery should be dissected [55]. As reported in Perrakis et al., the transverse colon together with both flexures should have infrapancreatic lymph nodes as well as lymph nodes along the gastroepiploic arcade as regional lymph node stations [57]. This same idea should be debated in cases of gastric cancer and a mesogastric excision. A standard D2 lymphadenectomy is based on the JCOG9501 randomized trial that compared D2 with D2 plus a para-aortic lymphadenectomy. For tumours located in the lower third of the stomach for a D2 lymphadenectomy, stations number 13 (retropancreatic) and 14v along the superior mesenteric vein are a part of the lymphadenectomy [42]. Based on series of papers from the Italian Gastric Cancer Research Group (GIRCG), which include being located along the edge of the organ and encompassing some of the main blood vessels that connect them to the main organ [58,59,60,61], special attention should also be paid to the complete removal of infrapyloric lymph nodes (station 6) as seen in distal third gastric cancers with station number 6 involvement. Furthermore, even in early forms, a resection of station number 14v is advised by some authors [62]. Interestingly, we have also proposed the possibility of a tailored lymphadenectomy, not only according to the tumour position and Lauren histotype but also based on the molecular classification of the gastric cancer [60]. The possible role of implementing these factors in a tailored lymphadenectomy in cases with mesogastric excisions needs to be evaluated.

## 6. Conclusions

Mesogastric excisions are still a technique that needs closer attention and further scientific evidence. From a technical point of view, the technique may be feasibly implemented in all types of operations with a curative intent. Additionally, not only classical open approaches but also laparoscopic approaches have been documented. Benefits of this technique from the literature seem to be possible; however, we need to wait for overall survival benefit studies. The same history was a part of the discussion concerning total mesorectal and mesocolic excisions, and today, both techniques are routine operations in many hospitals worldwide. Future studies are needed, and ongoing clinical trials may provide the first answers regarding the direction that the story concerning the mesogastrium will follow.

## Figures and Tables

**Table 1:** Scoring of modified mesogastrium—modified proposition by Xie et al. of analysing the quality of CME by scoring different parameters by surgical, pathological and embryo-anatomical factors. With “6” being the highest mark while “0” indicated the poorest sample.

Scoring of Mesogastrium						
Surgery and Histopatological Scoring of Mesogastrium	“Tri-Junction”	“Pumpkin-Like Surface”	“Little Square”	Root Ligation	Bleeding Amount	Lymph Nodes of Mesogastrium
**0 point**	Failed to find	Failed to expose	Failed to expose	Failed to reach the root part of blood vessels	>40 mL	Organ + Lymphatic stations 1–6
**0.5 point**	Not obvious	Not obvious	Not obvious	Not quite satisfied	20–40 mL	Lymphatic stations 1–6 + 7, 8, 10–12
**1 point**	Very obvious	Very obvious	Very obvious	Quite satisfied	<20 mL	Lymphatic stations 1–8, 10–12 + 9
